# Genome Sequence of a Presumptive *Mannheimia haemolytica* Strain with an A1/A6-Cross-Reactive Serotype from a White-Tailed Deer (*Odocoileus virginianus*)

**DOI:** 10.1128/genomeA.00114-14

**Published:** 2014-03-27

**Authors:** Paulraj K. Lawrence, Russell F. Bey, Brittanny Wiener, Weerayuth Kittichotirat, Roger E. Bumgarner

**Affiliations:** a Newport Laboratories Ltd., Worthington, Minnesota, USA; b Systems Biology and Bioinformatics Research Group, Pilot Plant, Development and Training Institute, King Mongkut’s University of Technology Thonburi, Bangkhuntien, Bangkok, Thailand; c Department of Microbiology, University of Washington, Seattle, Washington, USA

## Abstract

*Mannheimia haemolytica* is a Gram-negative bacterium and the principal etiological agent associated mostly with bovine respiratory disease complex. However, we report here the sequence of a strain with the novel A1/A6-cross-reactive serotype, strain PKL10, isolated from white-tailed deer. PKL10 was isolated from the spleen of farmed white-tailed deer showing clinical signs of pneumonia. The genome structure of PKL10 is dramatically different from that of previously sequenced isolates, which was demonstrated by genome alignments. In addition, the coding sequences in PKL10 share approximately 86% sequence identity with the coding sequences in other fully sequenced *M. haemolytica* strains. This suggests that PKL10 is a novel *Mannheimia* species.

## GENOME ANNOUNCEMENT

*Mannheimia haemolytica* is a beta-hemolytic Gram-negative bacterium and the principal etiological agent associated with respiratory disease complex in ruminants. *M. haemolytica* is a normal commensal of the upper respiratory tract and tonsillar crypts in healthy ruminants. However, in the case of animals with compromised pulmonary defense mechanisms resulting from viral infections and stress, *M. haemolytica* bacteria migrate into the lungs, causing acute fibrinous pleuropneumonia or pasteurellosis, commonly known as “shipping fever” (1–3). The global cattle industry suffers substantial losses due to *M. haemolytica*-induced pneumonia, accounting for almost 30% of the total cattle deaths worldwide (4–6). The U.S. cattle industry loses more than $1 billion annually to shipping fever or bovine respiratory disease complex (7). In addition, *M. haemolytica*-induced pneumonia decimates other domestic and wild ruminants, including farm-raised white-tailed deer (*Odocoileus virginianus*) (3, 8). Bronchopneumonia is the major cause of death in farm-raised white-tailed deer (8). Furthermore, *Fusobacterium necrophorum* and *M. haemolytica* are the major repeats in toxin (RTX)-producing pathogens isolated postmortem (8). Here, we report the genome sequencing of a presumptive *M. haemolytica* isolate (strain PKL10) that was cultured from the spleen of a white-tailed deer showing clinical signs of pneumonia. This isolate cross-reacts equally with A1 and A6 antisera raised in rabbits.

The complete genome sequence of strain PKL10 was determined via Illumina sequencing using paired-end 250-bp reads. Assembly was performed using the Geneious assembler (Biomatters Ltd.), which generated 11 large (>1-kb) contigs, with an average coverage of 108×. The 11 large contigs from PKL10 sum 2,324,226 bp and contain 2,771 predicted coding sequences, 64 tRNA genes, and 20 rRNA genes. This compares to an average genome size of 2,655,124 bp and an average of 2,640 coding sequences based on the five complete *M. haemolytica* sequences presently in GenBank (accession no. CP004752, CP005383, CP005972, CP006573, and CP006574).

To assess the genome structure of PKL10, we performed alignments of all contigs against the completed genome with accession no. CP004752 using the whole-genome alignment program Mauve (9). The average length of a locally collinear block (LCB) in PKL10 relative to that of CP004752 is 12.6 kbp. For comparison, we performed pairwise Mauve whole-genome alignments between all fully completed *M. haemolytica* genomes. In these alignments, the average length of an LCB is 121.8 kbp. This is about 10× larger than what is seen in a comparison of PKL10 and CP004752, indicating an extensive genomic rearrangement of PKL10 relative to the *M. haemolytica* genomes. Analysis of the leukotoxin operon genes *lktCABD* showed that PKL10 shares only 80 to 88% homology with the five completely sequenced *M. haemolytica* isolates, which share 99 to 100% homology between them. The housekeeping genes *dnaJ* and *rpoB* from PKL10 are only 94 and 91% identical to those of the known *M. haemolytica* isolates, respectively. When coding sequences of PKL10 are used to search GenBank, the most frequent best BLAST hit is *M. haemolytica*; however, the average sequence similarity is only 86%. Taken together, these results indicate that PKL10 is likely a previously unknown species of *Mannheimia* that is capable of infecting the *Cervidae* family.

### Nucleotide sequence accession numbers.

The whole-genome shotgun project for *M. haemolytica* strain PKL10 has been deposited at DDBJ/EMBL/GenBank under the accession no. JANJ00000000. The version described in this paper is version JANJ01000000.
